# Supplementary material for: Transcriptomic analysis of insecticide resistance in the lymphatic filariasis vector Culex quinquefasciatus
Source: Sci Rep. 2019 Aug 6;9:11406. doi: 10.1038/s41598-019-47850-w (PMC6684662; doi:10.1038/s41598-019-47850-w)
Supplement: Supplementary file 1 — Supplementary material 1 [file 41598_2019_47850_MOESM1_ESM.pdf]

Supplementary information

Transcriptomic analysis of insecticide resistance in the lymphatic filariasis vector Culex quinquefasciatus

Walter Fabricio Silva Martins*,+,1,2, Craig Stephen Wilding+,1,3, Alison Taylor Isaacs1, Emily Joy Rippon1, Karine Megy4,5, Martin James Donnelly1,6

1 Department of Vector Biology, Liverpool School of Tropical Medicine , Liverpool, UK, 2 Universidade Estadual da Paraíba, Campina Grande, Brasil, 3 School of Natural Sciences and Psychology, Liverpool John Moores University, Liverpool, UK, 4European Bioinformatics Institute (EMBL-EBI), Wellcome Trust Genome Campus, Hinxton, UK 5 Current address: Department of Haematology, University of Cambridge & NHS Blood and Transplant, Cambridge, UK, 6Malaria Programme, Wellcome Trust Sanger Institute, Hinxton, UK. +These authors contributed equally to this work.
*Correspondence to fabricio.martins@lstmed.ac.uk

```
# This output was generated with AUGUSTUS (version 2.7).
# AUGUSTUS is a gene prediction tool for eukaryotes written by Mario Stanke
(mario.stanke@uni-greifswald.de)
# and Oliver Keller (keller@cs.uni-goettingen.de).
# Please cite: Mario Stanke, Mark Diekhans, Robert Baertsch, David Haussler
(2008),
# Using native and syntenically mapped cDNA alignments to improve de novo gene
finding
# Bioinformatics 24: 637-644, doi 10.1093/bioinformatics/btn013
# No extrinsic information on sequences given.
# Initialising the parameters ...
# aedes version. Using default transition matrix.
# Looks like /data/www/augustus/tmp/AUG-166261241/input.fa is in fasta format.
# We have hints for 0 sequences and for 0 of the sequences in the input set.
#
# ----- prediction on sequence number 1 (length = 4735, name = P450) -----
#
# Constraints/Hints:
# (none)
# Predicted genes for sequence number 1 on both strands
# start gene g1
P450    AUGUSTUS        gene    209     1747    0.94    -       .       g1
P450    AUGUSTUS        transcript      209     1747    0.94    -       .
g1.t1
P450    AUGUSTUS        stop_codon      209     211     .       -       0
transcript_id "g1.t1"; gene_id "g1";
P450    AUGUSTUS        terminal        209     381     0.98    -       2
transcript_id "g1.t1"; gene_id "g1";
P450    AUGUSTUS        initial 445     1747    0.96    -       0
transcript_id "g1.t1"; gene_id "g1";
P450    AUGUSTUS        intron  382     444     1       -       .
transcript_id "g1.t1"; gene_id "g1";
P450    AUGUSTUS        CDS     209     381     0.98    -       2
transcript_id "g1.t1"; gene_id "g1";
P450    AUGUSTUS        CDS     445     1747    0.96    -       0
```

```
transcript_id "g1.t1"; gene_id "g1";
P450    AUGUSTUS        start_codon     1745    1747    .       -       0
transcript_id "g1.t1"; gene_id "g1";
# coding sequence =
[atgatcatttactcgctgcttctcatcggtacctcgatctacctgatcctccggtacatctactcgtactgggatcgcc
#
atggcctgccgaacctcaaaccggacatcccgttcgggaacatccgtgccgtcgccctcaagcaggaatcgttcggcgtc
gccctgaacgctctccac
#
gccaaaaccacaggccaactggtcggaatctacctactcttccgcccggcgatcctaatccgggacgcccacctagccca
ccgcatcataacgtccga
#
cttcaactacttccacgaccgcggtgtgcactgtgacgagagctcggatcccttttcggcgcacctgtttgccctgcccg
ggaagaggtggcgcagcc
#
tgaggaacaagctgactccgaccttcaccgctgggcagctgcgcggtatgctgccgacgatcttggccgttgggaggaag
tttcagggtttcttggaa
#
cccaaggcgaagcgaggggaggtgattgaggccagggatttgatatcgcgctttgtgctggagatcgtggcgtcggtgtt
tttcggttacgagatcaa
#
ctcgattcacgatccgcaggattcgtttcggacggttttacgatcatttcgggaggacaatcacgtcacaaatttgagaa
cggttggtgcgtttttgt
#
gtccgacgttgctgaaggtcagccgggtcaaaacggtacctgaggtggtggacaattttgtaaacaaaatcattagggag
cagatcgagtttcgcgag
#
aagaacaacgttacgaggaaggatttcatccaactgttgatcgatctccgacgggagaagagtgatttcggactttcgtt
ggaacagtgcgcggccaa
#
tgtgtttttgttctacgtagcaggagcggatacctcaacggatgccatcacctacacggtccacgagctgacccatcgac
cggatcttatgaagaagg
#
ttcaagcagagatcgacgatgcgcttgagaagtccaacggtgaaatcaactacgatgtactccacgaaatgaaactttta
gacaactgcgtgaaggaa
#
acccttagaaagtacccgttcccaattttaaatcgcgagtgtacccaggactatcaggttccagattcgaagctgatcat
caggaagggaactccggt
#
gatcataccgctgcaagcgttcggaatgagtgaggagtacttcccggaacctaatcgctacctacctgaacgatttgatt
catccacaaagaattacg
#
acgaaaaagcttacattccatttggagatggtccgaggaattgtattggttcccggatgggcagtgccgtttcgaagatc
ggcatcatcatgctgctt
#
tcgaagttcaactttgaggcgactcaaggtgcggagataggctttgctcgggcccaaattgcgctggctccggagaatgg
catttcacttaagatttc
# caacaggataagaaactcgatataa]
# protein sequence =
[MIIYSLLLIGTSIYLILRYIYSYWDRHGLPNLKPDIPFGNIRAVALKQESFGVALNALHAKTTGQLVGIYLLFRPAIL
#
IRDAHLAHRIITSDFNYFHDRGVHCDESSDPFSAHLFALPGKRWRSLRNKLTPTFTAGQLRGMLPTILAVGRKFQGFLEP
```

KAKRGEVIEARDLISRFV
#
LEIVASVFFGYEINSIHDPQDSFRTVLRSFREDNHVTNLRTVGAFLCPTLLKVSRVKTVPEVVDNFVNKIIREQIEFREK
NNVTRKDFIQLLIDLRRE
#
KSDFGLSLEQCAANVFLFYVAGADTSTDAITYTVHELTHRPDLMKKVQAEIDDALEKSNGEINYDVLHEMKLLDNCVKET
LRKYPFPILNRECTQDYQ
#
VPDSKLIIRKGTPVIIPLQAFGMSEEYFPEPNRYLPERFDSSTKNYDEKAYIPFGDGPRNCIGSRMGSAVSKIGIIMLLS
KFNFEATQGAEIGFARAQ
IALAPENGISLKISNRIRNSI]
end gene g1
###
start gene g2
P450 AUGUSTUS gene 3848 4639 0.69 - . g2
P450 AUGUSTUS transcript 3848 4639 0.69 - .
g2.t1
P450 AUGUSTUS stop_codon 3848 3850 . - 0
transcript_id "g2.t1"; gene_id "g2";
P450 AUGUSTUS terminal 3848 4014 0.96 - 2
transcript_id "g2.t1"; gene_id "g2";
P450 AUGUSTUS initial 4072 4639 0.73 - 0
transcript_id "g2.t1"; gene_id "g2";
P450 AUGUSTUS intron 4015 4071 0.99 - .
transcript_id "g2.t1"; gene_id "g2";
P450 AUGUSTUS CDS 3848 4014 0.96 - 2
transcript_id "g2.t1"; gene_id "g2";
P450 AUGUSTUS CDS 4072 4639 0.73 - 0
transcript_id "g2.t1"; gene_id "g2";
P450 AUGUSTUS start_codon 4637 4639 . - 0
transcript_id "g2.t1"; gene_id "g2";
coding sequence =
[atgaccaacctagcgacgcagcagatgaagtatcgtgagaagaatgacttggctagaaaggatttcttgcagttgctga
#
atgatcttcaccaagttgatttgtcagctgaagagtgcgcatcgaatgtgaatctgttctacactgcaggttcggaaacc
accaaatctacagtcatc
#
tatactcttcacgaactagctcaccatccagaagttatgagacggcttgtggaagaagttgatgaatacgtcaagcaatc
aggtggtgagattagcta
#
cgatcttgttaagagtatgccatatttggacctgtgcgtgaaggaaaccctgagaaagtatcccggactgtttttcctga
accggaagtgcacccacg
#
actataaggttcccaactctcggctggtcatcaaaaagggtacccaaataattatcccgtcgatggcctacggcatggat
gagcggtgtttcccgaat
#
ccggagagctacatccccgaacgatttcttgaggagaccaaaaattacgacgaggacgcctacgcaccgtttggagaagg
accgcggaagtgtatcgc
#
tcctcgaatgggaattttcgtcgccaaagtgaccttggtgaggctgctgtccaagtttcggttcgaggctacgcaagagc
tgaaggttgagtttgccc
cctcggtgattccgctcgtgccgaaggatggagtcaggatgaagattcacaaaagaagtgtttggtaa]

```
# protein sequence =
[MTNLATQQMKYREKNDLARKDFLQLLNDLHQVDLSAEECASNVNLFYTAGSETTKSTVIYTLHELAHHPEVMRRLVEE
#
VDEYVKQSGGEISYDLVKSMPYLDLCVKETLRKYPGLFFLNRKCTHDYKVPNSRLVIKKGTQIIIPSMAYGMDERCFPNP
ESYIPERFLEETKNYDED
# AYAPFGEGPRKCIAPRMGIFVAKVTLVRLLSKFRFEATQELKVEFAPSVIPLVPKDGVRMKIHKRSVW]
# end gene g2
###
```
