# Supplementary material for: Transcriptomic analysis of insecticide resistance in the lymphatic filariasis vector Culex quinquefasciatus
Source: Sci Rep. 2019 Aug 6;9:11406. doi: 10.1038/s41598-019-47850-w (PMC6684662; doi:10.1038/s41598-019-47850-w)
Supplement: Supplementary file 2 — Supplementary Figures [file 41598_2019_47850_MOESM2_ESM.pdf]

Transcriptomic analysis of insecticide resistance in the lymphatic filariasis vector *Culex quinquefasciatus*

Walter Fabricio Silva Martins*[,+,1,2], Craig Stephen Wilding[+,1,3], Alison Taylor Isaacs[1], Emily Joy Rippon[1], Karine Megy[4,5], Martin James Donnelly[1,6]

[1] Department of Vector Biology, Liverpool School of Tropical Medicine , Liverpool, UK, [2] *Universidade Estadual da Paraíba, Campina Grande, Brasil,* [3] *School of Natural Sciences and Psychology, Liverpool John Moores University, Liverpool, UK,* [4]*European Bioinformatics Institute (EMBL-EBI), Wellcome Trust Genome Campus, Hinxton, UK* [5] *Current address: Department of Haematology, University of Cambridge & NHS Blood and Transplant, Cambridge, UK,* [6]Malaria Programme, Wellcome Trust Sanger Institute, *Hinxton*, UK. [+]These authors contributed equally to this work.

*Correspondence to fabricio.martins@lstmed.ac.uk

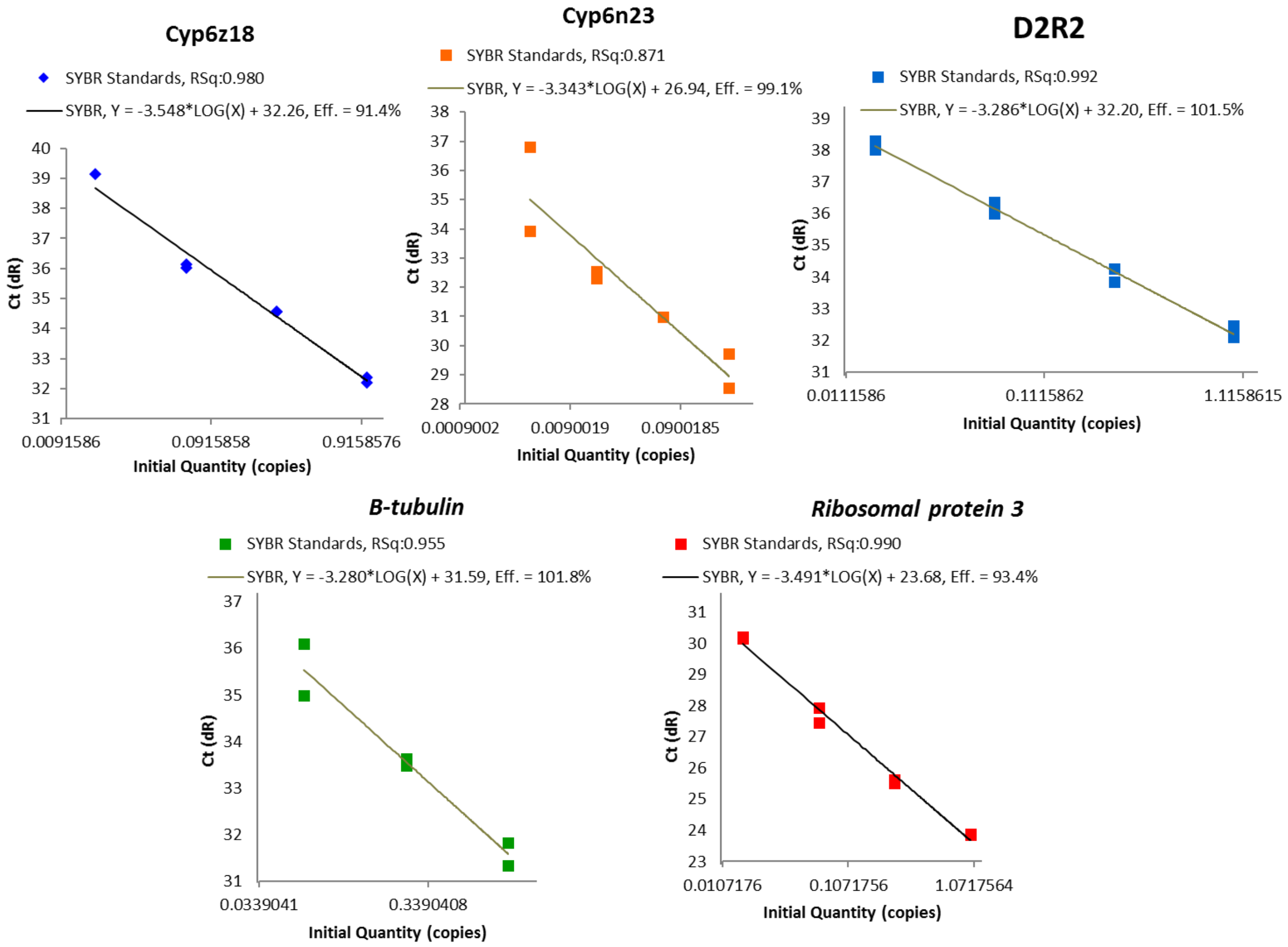

Figure S1: Standard curve from primers used on real-time PCR for microarray candidate genes validation. Squares correspond to 1x10 serial dilution of cDNA. qPCR efficiency (Eff) and coefficient of determination (Rsq) were calculated for each prime primer based two replicated.

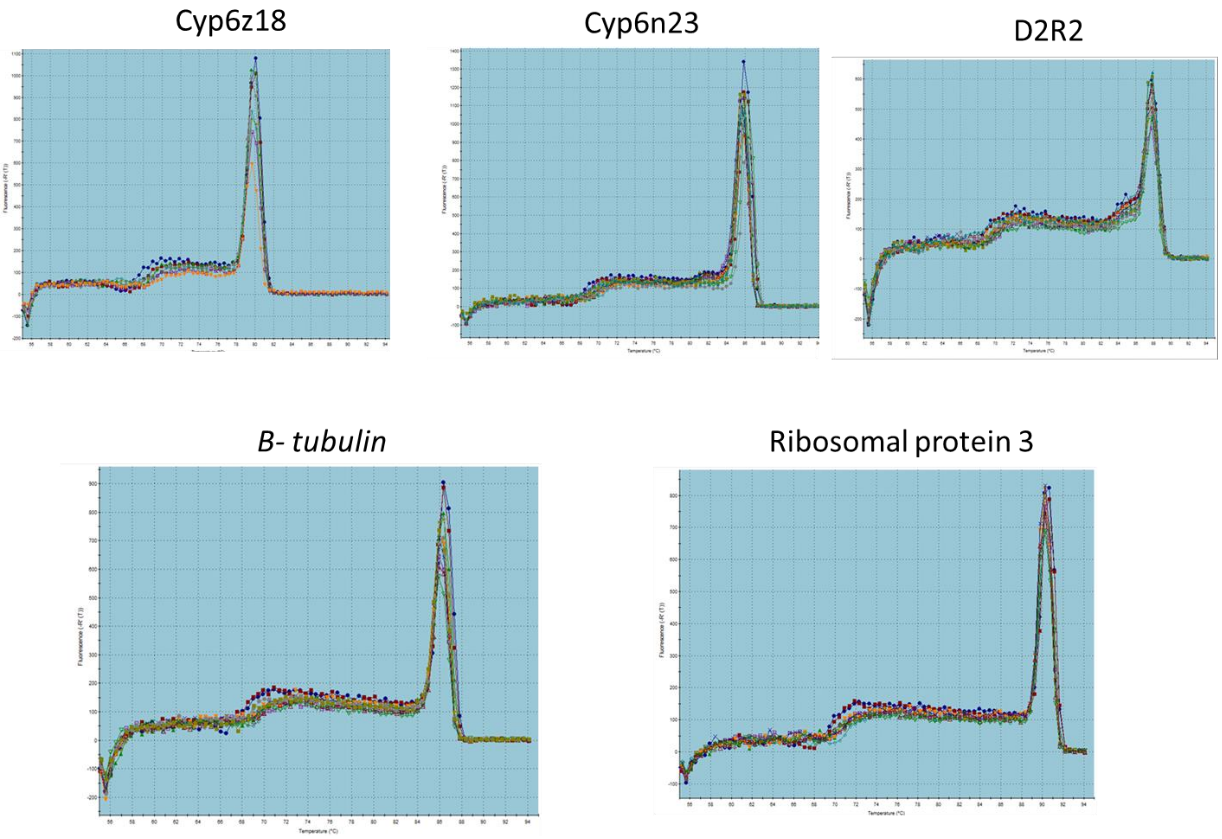

Figure S2: Validation of qPCR primers. Dissociation curves of real-time PCR amplification of microarray top candidate genes and endogenous control.

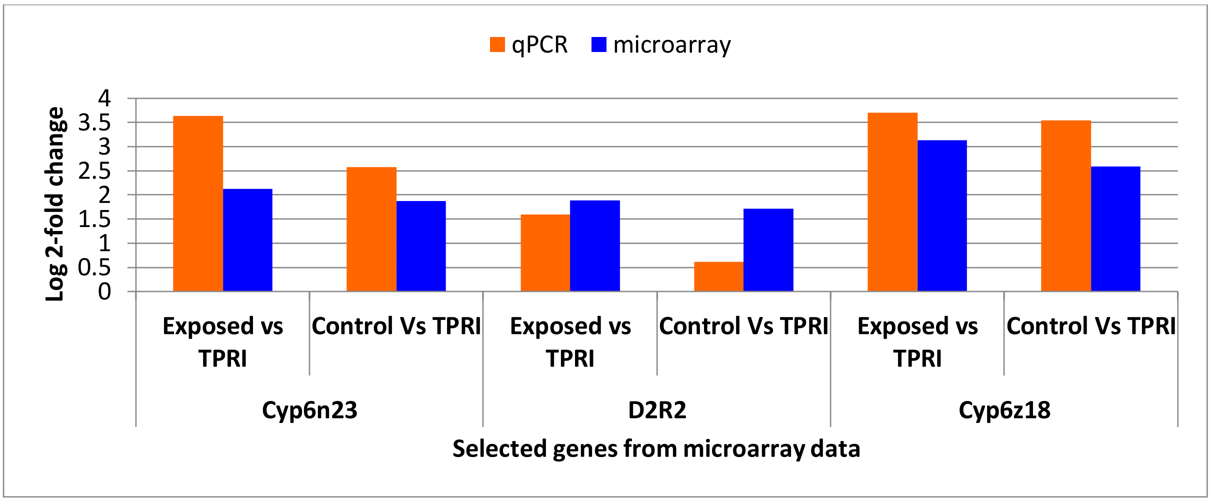

Figure S3: Pairwise comparison of microarray and qPCR data based on gene expression profile for three genes found to be significantly different expressed between Uganda exposed and non-exposed mosquitoes (sympatric control) and TPRI susceptible strain
