# Supplementary material for: Transcriptomic analysis of insecticide resistance in the lymphatic filariasis vector Culex quinquefasciatus
Source: Sci Rep. 2019 Aug 6;9:11406. doi: 10.1038/s41598-019-47850-w (PMC6684662; doi:10.1038/s41598-019-47850-w)
Supplement: Supplementary file 3 — Supplementary Table S1 and S2 [file 41598_2019_47850_MOESM3_ESM.pdf]

Transcriptomic analysis of insecticide resistance in the lymphatic filariasis vector *Culex quinquefasciatus*

Walter Fabricio Silva Martins*[,+,1,2], Craig Stephen Wilding[+,1,3], Alison Taylor Isaacs[1], Emily Joy Rippon[1], Karine Megy[4,5], Martin James Donnelly[1,6]

[1] Department of Vector Biology, Liverpool School of Tropical Medicine, Liverpool, UK, [2] Universidade Estadual da Paraíba, Campina Grande, Brasil, [3] School of Natural Sciences and Psychology, Liverpool John Moores University, Liverpool, UK, [4]European Bioinformatics Institute (EMBL-EBI), Wellcome Trust Genome Campus, Hinxton, UK [5] Current address: Department of Haematology, University of Cambridge & NHS Blood and Transplant, Cambridge, UK, [6]Malaria Programme, Wellcome Trust Sanger Institute, Hinxton, *UK*. [+]These authors contributed equally to this work.

*Correspondence to fabricio.martins@lstmed.ac.uk

Table S1: PCR primers used for qPCR gene expression analysis

Gene name	Sequence (5`-3`)	Fragment size (bp)	Accession number[A]
Cyp6z18	F: TAATCGCTACCTACCTGAACGA<br>R: AATCGTTCAGGTAGGTAGCG	107	CPIJ020018-RA
Cyp6n23	F: CCGGAAGAGCTCGCCAAA<br>R: GCGTCAATCCGTAGCGGT	176	CPIJ005900-RA
D2R2	F: TTCGAGGTTCCAGAGTTTGAG<br>R: GCATCGCTTGCAGTGTTT	169	CPIJ011746-RA
β-tubulin	F: TCGACCTTCATCGGAAACAC<br>R: GTTCATGTTGCTCTCAGCCT	156	CPIJ003263-RA
40S ribosomal protein S3	F: CCTGACTCGCGAGCTGG<br>R: GAAACCGAATCGCTTCTGGAC	166	CPIJ013941-RA

[A] VectorBase transcript identification

Table S2: Primer sequences for amplification and sequencing of the *Cyp6z16* genomic region

Primer ID	Sequence (5`-3`)
	**PCR primers**
Cx_6Z16-F	AAAGGTGAACTGAGGGCAAA
Cx_6Z16-R	CTGATAAACAACGTTCGGACA
	**Sequencing primers**
Cx_6Z16-seq2	TGGAGGTGAATGCGAAAAGT
Cx_6Z16-seq3	TTTCATTTCGTGGAGTACATCG
Cx_6Z16-seq4	ATGGCATCCGTTGAGGTATC
Cx_6Z16-seq5	TCGAGTACCGATGAGAAGCA
Cx_6Z16-seq6	CGGCTGATTTCAACCATTTT
Cx_6Z16-seq7	TTGAAATGTTTTAGGGGAGCA
Cx_6Z16-seq8	TTTCCGATCTCTTCGCAAAC
Cx_6Z16-seq9	ATAGTCGTGGGTGCACTTCC
