# Supplementary material for: Transcriptomic analysis of insecticide resistance in the lymphatic filariasis vector Culex quinquefasciatus
Source: Sci Rep. 2019 Aug 6;9:11406. doi: 10.1038/s41598-019-47850-w (PMC6684662; doi:10.1038/s41598-019-47850-w)
Supplement: Supplementary file 6 — dataset 3 [file 41598_2019_47850_MOESM6_ESM.pdf]

Transcriptomic analysis of insecticide resistance in the lymphatic filariasis vector *Culex quinquefasciatus*

Walter Fabricio Silva Martins[*,+,1,2], Craig Stephen Wilding[+,1,3], Alison Taylor Isaacs[1], Emily Joy Rippon[1], Karine Megy[4,5], Martin James Donnelly[1,6]

[1] Department of Vector Biology, Liverpool School of Tropical Medicine , Liverpool, UK, [2] *Universidade Estadual da Paraíba, Campina Grande, Brasil,* [3] *School of Natural Sciences and Psychology, Liverpool John Moores University, Liverpool, UK,* [4]*European Bioinformatics Institute (EMBL-EBI), Wellcome Trust Genome Campus, Hinxton, UK* [5] *Current address: Department of Haematology, University of Cambridge & NHS Blood and Transplant, Cambridge, UK,* [6]Malaria Programme, Wellcome Trust Sanger Institute, *Hinxton, UK.* [+]These authors contributed equally to this work.

*Correspondence to fabricio.martins@lstmed.ac.uk

Table S5. Clusters annotation based on enrichment analysis results from a list of probes with a -log10 false-discovery rate (FDR) > 2 obtained from the ANOVA

**Annotation Cluster 1 - Enrichment Score: 5.15**					
**Database**	**GO term name**		**Count**	**P-Value**	**Benjamini**
INTERPRO	Glutathione S-transferase, N-terminal	RT	10	1.50E-08	6.70E-06
INTERPRO	Glutathione S-transferase, C-terminal-like	RT	10	1.20E-07	2.80E-05
INTERPRO	Glutathione S-transferase, C-terminal	RT	9	1.30E-07	2.00E-05
INTERPRO	Thioredoxin-like fold	RT	10	1.10E-04	1.30E-02
GOTERM_MF_DIRECT	transferase activity	RT	9	1.50E-03	1.70E-01
COG_ONTOLOGY	Posttranslational modification, protein turnover, chaperones	RT	10	2.90E-03	4.90E-02
**Annotation Cluster 2 - Enrichment Score: 1.9**					
UP_KEYWORDS	Chromosome	RT	8	1.80E-03	9.40E-02
SMART	H3	RT	5	2.00E-03	1.70E-01
INTERPRO	Histone core	RT	7	2.00E-03	1.40E-01
INTERPRO	Histone H3	RT	5	2.10E-03	1.30E-01
UP_KEYWORDS	Nucleus	RT	16	2.70E-03	9.10E-02
INTERPRO	Histone-fold	RT	8	5.20E-03	2.30E-01
GOTERM_CC_DIRECT	nucleosome	RT	7	2.90E-02	4.20E-01
UP_KEYWORDS	Nucleosome core	RT	4	1.40E-01	5.90E-01
UP_KEYWORDS	DNA-binding	RT	8	2.00E-01	6.70E-01
GOTERM_MF_DIRECT	DNA binding	RT	12	5.50E-01	1.00E+00
**Annotation Cluster 3 - Enrichment Score: 1.47**					
GOTERM_MF_DIRECT	NAD binding	RT	4	4.60E-03	2.50E-01
KEGG_PATHWAY	Carbon metabolism	RT	8	4.90E-03	2.70E-01
KEGG_PATHWAY	2-Oxocarboxylic acid metabolism	RT	3	3.90E-02	7.10E-01

KEGG_PATHWAY	Biosynthesis of amino acids	RT	5	4.20E-02	5.90E-01
UP_KEYWORDS	Oxidoreductase	RT	9	8.50E-02	5.20E-01
KEGG_PATHWAY	Biosynthesis of antibiotics	RT	8	9.70E-02	6.60E-01
KEGG_PATHWAY	Citrate cycle (TCA cycle)	RT	3	1.70E-01	7.70E-01
**Annotation Cluster 4 - Enrichment Score: 1.45**					
INTERPRO	Peptidase S1, trypsin family, active site	RT	11	4.50E-03	2.30E-01
INTERPRO	Peptidase S1A, chymotrypsin-type	RT	13	1.10E-02	4.00E-01
UP_KEYWORDS	Disulfide bond	RT	15	2.00E-02	4.20E-01
SMART	Tryp_SPc	RT	13	4.10E-02	7.30E-01
INTERPRO	Peptidase S1	RT	13	4.70E-02	8.20E-01
UP_KEYWORDS	Serine protease	RT	6	5.00E-02	4.60E-01
INTERPRO	Trypsin-like cysteine/serine peptidase domain	RT	13	5.40E-02	7.90E-01
UP_KEYWORDS	Protease	RT	12	1.00E-01	5.40E-01
GOTERM_MF_DIRECT	serine-type endopeptidase activity	RT	13	1.60E-01	9.70E-01
**Annotation Cluster 5 - Enrichment Score: 1.43**					
GOTERM_CC_DIRECT	mitochondrial inner membrane	RT	4	2.10E-02	5.40E-01
UP_KEYWORDS	Mitochondrion	RT	5	3.40E-02	4.10E-01
UP_KEYWORDS	Mitochondrion inner membrane	RT	3	7.40E-02	5.30E-01
**Annotation Cluster 6 - Enrichment Score: 1.13**					
UP_KEYWORDS	Ribosomal protein	RT	10	3.20E-02	4.40E-01
KEGG_PATHWAY	Ribosome	RT	8	5.10E-02	5.60E-01
UP_KEYWORDS	Ribonucleoprotein	RT	10	7.10E-02	5.50E-01
GOTERM_CC_DIRECT	ribosome	RT	10	1.00E-01	7.30E-01
GOTERM_BP_DIRECT	translation	RT	9	1.10E-01	9.90E-01

GOTERM_MF_DIRECT	structural constituent of ribosome	RT	9	1.30E-01	9.70E-01
**Annotation Cluster 7 - Enrichment Score: 1.03**					
INTERPRO	Mitochondrial substrate/solute carrier	RT	4	5.10E-02	8.00E-01
INTERPRO	Mitochondrial carrier domain	RT	4	5.10E-02	8.00E-01
UP_KEYWORDS	Transport	RT	8	3.00E-01	7.40E-01
**Annotation Cluster 8 - Enrichment Score: 0.94**					
UP_KEYWORDS	Serine protease	RT	6	5.00E-02	4.60E-01
SMART	CLIP	RT	3	1.40E-01	9.70E-01
INTERPRO	Proteinase, regulatory CLIP domain	RT	3	2.00E-01	9.90E-01
**Annotation Cluster 9 - Enrichment Score: 0.74**					
UP_KEYWORDS	Protein biosynthesis	RT	6	7.80E-02	5.20E-01
UP_KEYWORDS	Elongation factor	RT	3	2.10E-01	6.70E-01
GOTERM_MF_DIRECT	translation elongation factor activity	RT	3	3.60E-01	1.00E+00
**Annotation Cluster 10 - Enrichment Score: 0.69**					
INTERPRO	Elongation factor, GTP-binding domain	RT	3	3.70E-02	7.60E-01
INTERPRO	Translation elongation/initiation factor/Ribosomal, beta-barrel	RT	3	1.90E-01	9.90E-01
GOTERM_MF_DIRECT	GTPase activity	RT	3	3.60E-01	1.00E+00
GOTERM_MF_DIRECT	GTP binding	RT	4	6.70E-01	1.00E+00
**Annotation Cluster 11 - Enrichment Score: 0.66**					
SMART	S_TK_X	RT	3	2.80E-02	7.30E-01
INTERPRO	AGC-kinase, C-terminal	RT	3	4.90E-02	8.10E-01
UP_KEYWORDS	Serine/threonine-protein kinase	RT	3	2.80E-01	7.30E-01

INTERPRO	Protein kinase, catalytic domain	RT	5	5.70E-01	1.00E+00
GOTERM_MF_DIRECT	protein serine/threonine kinase activity	RT	3	5.80E-01	1.00E+00
INTERPRO	Protein kinase-like domain	RT	5	8.30E-01	1.00E+00
**Annotation Cluster 12 - Enrichment Score: 0.58**					
UP_KEYWORDS	ATP-binding	RT	11	1.30E-01	6.10E-01
UP_KEYWORDS	Nucleotide-binding	RT	12	2.20E-01	6.50E-01
GOTERM_MF_DIRECT	ATP binding	RT	19	6.30E-01	1.00E+00
**Annotation Cluster 13 - Enrichment Score: 0.55**					
UP_KEYWORDS	FAD	RT	3	2.00E-01	6.80E-01
UP_KEYWORDS	Flavoprotein	RT	3	2.20E-01	6.60E-01
GOTERM_MF_DIRECT	flavin adenine dinucleotide binding	RT	3	5.20E-01	1.00E+00
**Annotation Cluster 14 - Enrichment Score: 0.43**					
INTERPRO	Glycoside hydrolase, catalytic domain	RT	4	2.00E-01	9.90E-01
INTERPRO	Glycoside hydrolase, superfamily	RT	4	2.90E-01	1.00E+00
GOTERM_BP_DIRECT	carbohydrate metabolic process	RT	3	8.80E-01	1.00E+00
**Annotation Cluster 15 - Enrichment Score: 0.41**					
UP_KEYWORDS	Membrane	RT	54	1.70E-01	6.40E-01
UP_KEYWORDS	Transmembrane helix	RT	49	3.70E-01	7.90E-01
UP_KEYWORDS	Transmembrane	RT	49	3.90E-01	8.00E-01
GOTERM_CC_DIRECT	integral component of membrane	RT	49	9.80E-01	1.00E+00
**Annotation Cluster 16 - Enrichment Score: 0.37**					
INTERPRO	WD40 repeat	RT	6	3.10E-01	1.00E+00
INTERPRO	WD40/YVTN repeat-like-containing domain	RT	7	3.50E-01	1.00E+00

INTERPRO	WD40-repeat-containing domain	RT	6	4.80E-01	1.00E+00
SMART	WD40	RT	5	5.00E-01	1.00E+00
INTERPRO	WD40 repeat, conserved site	RT	3	5.60E-01	1.00E+00
**Annotation Cluster 17 - Enrichment Score: 0.34**					
INTERPRO	Zinc finger, RING-type, conserved site	RT	3	1.70E-01	9.90E-01
SMART	RING	RT	3	4.00E-01	1.00E+00
INTERPRO	Zinc finger, RING-type	RT	3	6.50E-01	1.00E+00
INTERPRO	Zinc finger, RING/FYVE/PHD-type	RT	3	9.30E-01	1.00E+00
**Annotation Cluster 18 - Enrichment Score: 0.33**					
INTERPRO	Protein kinase C-like, phorbol ester/diacylglycerol binding	RT	3	1.30E-01	9.70E-01
GOTERM_BP_DIRECT	intracellular signal transduction	RT	4	2.80E-01	1.00E+00
INTERPRO	Protein kinase, catalytic domain	RT	5	5.70E-01	1.00E+00
SMART	S_TKc	RT	3	7.30E-01	1.00E+00
GOTERM_CC_DIRECT	intracellular	RT	6	7.80E-01	1.00E+00
INTERPRO	Protein kinase-like domain	RT	5	8.30E-01	1.00E+00
**Annotation Cluster 19 - Enrichment Score: 0.25**					
UP_KEYWORDS	Zinc	RT	8	3.40E-01	7.80E-01
UP_KEYWORDS	Zinc-finger	RT	5	4.30E-01	8.40E-01
UP_KEYWORDS	Metal-binding	RT	10	6.60E-01	9.60E-01
GOTERM_MF_DIRECT	metal ion binding	RT	9	9.80E-01	1.00E+00
**Annotation Cluster 20 - Enrichment Score: 0.21**					
UP_KEYWORDS	ANK repeat	RT	3	5.00E-01	8.90E-01
SMART	ANK	RT	3	6.60E-01	1.00E+00
INTERPRO	Ankyrin repeat	RT	3	6.60E-01	1.00E+00

INTERPRO	Ankyrin repeat-containing domain	RT	3	6.80E-01	1.00E+00
**Annotation Cluster 21 - Enrichment Score: 0.15**					
INTERPRO	Nucleotide-binding, alpha-beta plait	RT	4	6.60E-01	1.00E+00
SMART	RRM	RT	3	6.80E-01	1.00E+00
GOTERM_MF_DIRECT	nucleotide binding	RT	5	7.20E-01	1.00E+00
INTERPRO	RNA recognition motif domain	RT	3	7.60E-01	1.00E+00
**Annotation Cluster 22 - Enrichment Score: 0.06**					
INTERPRO	Zinc finger, AD-type	RT	7	5.80E-01	1.00E+00
SMART	SM00868	RT	6	6.60E-01	1.00E+00
GOTERM_MF_DIRECT	zinc ion binding	RT	14	9.50E-01	1.00E+00
INTERPRO	Zinc finger, C2H2-like	RT	8	9.60E-01	1.00E+00
SMART	ZnF_C2H2	RT	8	9.70E-01	1.00E+00
INTERPRO	Zinc finger C2H2-type/integrase DNA-binding domain	RT	6	9.70E-01	1.00E+00
INTERPRO	Zinc finger, C2H2	RT	8	9.70E-01	1.00E+00
GOTERM_MF_DIRECT	nucleic acid binding	RT	14	9.70E-01	1.00E+00
