# Supplementary material for: Transcriptomic analysis of insecticide resistance in the lymphatic filariasis vector Culex quinquefasciatus
Source: Sci Rep. 2019 Aug 6;9:11406. doi: 10.1038/s41598-019-47850-w (PMC6684662; doi:10.1038/s41598-019-47850-w)
Supplement: Supplementary file 7 — Dataset 4 [file 41598_2019_47850_MOESM7_ESM.pdf]

Transcriptomic analysis of insecticide resistance in the lymphatic filariasis vector *Culex quinquefasciatus*

Walter Fabricio Silva Martins*,+,[1,2], Craig Stephen Wilding+,[1,3], Alison Taylor Isaacs[1], Emily Joy Rippon[1], Karine Megy[4,5], Martin James Donnelly[1,6]

[1] Department of Vector Biology, Liverpool School of Tropical Medicine , Liverpool, UK, [2] *Universidade Estadual da Paraíba, Campina Grande, Brasil,* [3] *School of Natural Sciences and Psychology, Liverpool John Moores University, Liverpool, UK,* [4]*European Bioinformatics Institute (EMBL-EBI), Wellcome Trust Genome Campus, Hinxton, UK* [5] *Current address: Department of Haematology, University of Cambridge & NHS Blood and Transplant, Cambridge, UK*, [6]Malaria Programme, Wellcome Trust Sanger Institute, *Hinxton, UK.* +These authors contributed equally to this work.

*Correspondence to fabricio.martins@lstmed.ac.uk

Table S6. Gene set annotation enrichment analysis results from a list of probes with a -log10 false-discovery rate (FDR) > 2 obtained from the ANOVA. Go Term highlight in yellow belongs to highest cluster enrichment score.

Category	Term	RT	Count	%	P-Value	Benjamini
INTERPRO	Glutathione S-transferase, N-terminal	RT	10	3.8	0.0000000150	0.0000067000
INTERPRO	Glutathione S-transferase, C-terminal-like	RT	10	3.8	0.0000001200	0.0000280000
INTERPRO	Glutathione S-transferase, C-terminal	RT	9	3.4	0.0000001300	0.0000200000
INTERPRO	Thioredoxin-like fold	RT	10	3.8	0.0001100000	0.0130000000
UP_KEYWORDS	Transferase	RT	29	10.9	0.0001800000	0.0190000000
GOTERM_MF_DIRECT	transferase activity	RT	9	3.4	0.0015000000	0.1700000000
UP_KEYWORDS	Chromosome	RT	8	3	0.0018000000	0.0940000000
INTERPRO	NAD(P)-binding domain	RT	12	4.5	0.0019000000	0.1600000000
SMART	H3	RT	5	1.9	0.0020000000	0.1700000000
INTERPRO	Histone core	RT	7	2.6	0.0020000000	0.1400000000
INTERPRO	Histone H3	RT	5	1.9	0.0021000000	0.1300000000
UP_KEYWORDS	Nucleus	RT	16	6	0.0027000000	0.0910000000
COG_ONTOLOGY	Posttranslational modification, protein turnover, chaperones	RT	10	3.8	0.0029000000	0.0490000000
INTERPRO	Peptidase S1, trypsin family, active site	RT	11	4.1	0.0045000000	0.2300000000
GOTERM_MF_DIRECT	NAD binding	RT	4	1.5	0.0046000000	0.2500000000
KEGG_PATHWAY	Carbon metabolism	RT	8	3	0.0049000000	0.2700000000
INTERPRO	Histone-fold	RT	8	3	0.0052000000	0.2300000000
INTERPRO	Peptidase S1A, chymotrypsin-type	RT	13	4.9	0.0110000000	0.4000000000
UP_KEYWORDS	Disulfide bond	RT	15	5.6	0.0200000000	0.4200000000
GOTERM_CC_DIRECT	mitochondrial inner membrane	RT	4	1.5	0.0210000000	0.5400000000
UP_KEYWORDS	Ligase	RT	8	3	0.0260000000	0.4400000000
SMART	S_TK_X	RT	3	1.1	0.0280000000	0.7300000000
GOTERM_MF_DIRECT	NADP binding	RT	3	1.1	0.0280000000	0.6800000000
GOTERM_CC_DIRECT	nucleosome	RT	7	2.6	0.0290000000	0.4200000000
UP_KEYWORDS	Ribosomal protein	RT	10	3.8	0.0320000000	0.4400000000
INTERPRO	Polynucleotide kinase 3 phosphatase	RT	2	0.8	0.0340000000	0.7600000000
INTERPRO	Isocitrate and isopropylmalate dehydrogenases family	RT	2	0.8	0.0340000000	0.7600000000
INTERPRO	Isocitrate dehydrogenase NAD-dependent	RT	2	0.8	0.0340000000	0.7600000000

INTERPRO	HAD-superfamily hydrolase, subfamily IIIA	RT	2	0.8	0.0340000000	0.7600000000
INTERPRO	Polynucleotide 3'-phosphatase	RT	2	0.8	0.0340000000	0.7600000000
UP_KEYWORDS	Mitochondrion	RT	5	1.9	0.0340000000	0.4100000000
INTERPRO	HAD-like domain	RT	4	1.5	0.0350000000	0.7400000000
INTERPRO	Elongation factor, GTP-binding domain	RT	3	1.1	0.0370000000	0.7400000000
KEGG_PATHWAY	2-Oxocarboxylic acid metabolism	RT	3	1.1	0.0390000000	0.7100000000
GOTERM_MF_DIRECT	isocitrate dehydrogenase (NAD+) activity	RT	2	0.8	0.0400000000	0.7200000000
SMART	Tryp_SPc	RT	13	4.9	0.0410000000	0.7300000000
KEGG_PATHWAY	Biosynthesis of amino acids	RT	5	1.9	0.0420000000	0.5900000000
UP_KEYWORDS	Hydrolase	RT	21	7.9	0.0470000000	0.4700000000
INTERPRO	Peptidase S1	RT	13	4.9	0.0470000000	0.7900000000
GOTERM_MF_DIRECT	ligase activity	RT	6	2.3	0.0480000000	0.7000000000
INTERPRO	AGC-kinase, C-terminal	RT	3	1.1	0.0490000000	0.7900000000
UP_KEYWORDS	Serine protease	RT	6	2.3	0.0500000000	0.4600000000
KEGG_PATHWAY	Ribosome	RT	8	3	0.0510000000	0.5600000000
INTERPRO	Mitochondrial carrier domain	RT	4	1.5	0.0510000000	0.7800000000
INTERPRO	Mitochondrial substrate/solute carrier	RT	4	1.5	0.0510000000	0.7800000000
INTERPRO	Trypsin-like cysteine/serine peptidase domain	RT	13	4.9	0.0540000000	0.7700000000
GOTERM_BP_DIRECT	mitotic chromosome condensation	RT	3	1.1	0.0590000000	0.9900000000
INTERPRO	Forkhead-associated (FHA) domain	RT	3	1.1	0.0630000000	0.8100000000
INTERPRO	NOPS	RT	2	0.8	0.0670000000	0.8100000000
INTERPRO	GHMP kinase N-terminal domain	RT	2	0.8	0.0670000000	0.8100000000
INTERPRO	Isopropylmalate dehydrogenase-like domain	RT	2	0.8	0.0670000000	0.8100000000
SMART	SM01329	RT	2	0.8	0.0690000000	0.8100000000
UP_KEYWORDS	Ribonucleoprotein	RT	10	3.8	0.0710000000	0.5500000000
UP_KEYWORDS	Mitochondrion inner membrane	RT	3	1.1	0.0740000000	0.5300000000
UP_KEYWORDS	Protein biosynthesis	RT	6	2.3	0.0780000000	0.5200000000
INTERPRO	SMAD/FHA domain	RT	3	1.1	0.0820000000	0.8600000000
UP_KEYWORDS	Oxidoreductase	RT	9	3.4	0.0850000000	0.5200000000
KEGG_PATHWAY	beta-Alanine metabolism	RT	3	1.1	0.0890000000	0.6900000000

UP_KEYWORDS	Mitosis	RT	2	0.8	0.0940000000	0.5300000000
UP_KEYWORDS	Kinase	RT	11	4.1	0.0960000000	0.5100000000
KEGG_PATHWAY	Biosynthesis of antibiotics	RT	8	3	0.0970000000	0.6600000000
INTERPRO	Mevalonate/galactokinase	RT	2	0.8	0.0980000000	0.9000000000
INTERPRO	SKP1 component, POZ	RT	2	0.8	0.0980000000	0.9000000000
INTERPRO	Aspartic peptidase	RT	2	0.8	0.0980000000	0.9000000000
